# Identifying characteristic features of community orientation among community health nurses in Fiji

**DOI:** 10.1002/nop2.305

**Published:** 2019-07-01

**Authors:** Sachiko Tanabe, Satoko Yanagisawa, Silina Waqa-Ledua, Mereani Tukana

**Affiliations:** ^1^ Faculty of Nursing, School of Health Science Fujita Health University Toyoake Aichi Japan; ^2^ Graduate School of Nursing and Health Aichi Prefectural University Nagoya Aichi Japan; ^3^ Fiji Nursing Council Suva Fiji; ^4^ Pacific Eye Institute Suva Fiji

**Keywords:** community/public health nurses, Fiji, interview, management of community activity, primary health care

## Abstract

**Aim:**

The present study aimed to identify detailed characteristics of community orientation among community health nurses in Fiji.

**Design:**

Qualitative descriptive design using semi‐structured individual interviews.

**Methods:**

Twenty participants including expert nurses, novice nurses, policymakers and representatives from communities were interviewed between July–August 2015 in Fiji. Data were analysed using descriptive qualitative methods.

**Results:**

Three main themes described in detail characteristics of community orientation among community health nurses in Fiji: Trusting Relationships, Commitment and Activity Management. Trusting Relationships and Commitment were interrelated and served as foundations for community orientation that promoted and facilitated Activity Management. Reflection and a sense of self‐accomplishment in the CHN experiences during Activity Management further strengthened Commitment and Trusting Relationships. Community orientation leads to superior understanding of community health needs, effective use of resources and increased community participation in activities. Such activity management will contribute to promote health beyond the individual, extending to entire communities.

## 1 INTRODUCTION

Evidence‐based health care has been widely recognized for its effectiveness in promoting community health, and the many concepts and models for such activities reflecting the different perspectives focus on management of information, resources and environment (Anderson & McFarlane, 2010; Green & Kreuter, 2005; Hanson, 1988; Kreuter, 1992). Community orientation (CO) is one concept originally adopted by hospital organizations in the United States to promote the effective provision of services based on community health needs (Institute of Medicine, 1998; Murphy, 1975; Starfield, Cassady, Nanda, Forrest, & Berk, 1998). Traditionally, hospitals have been the bastions of acute disease treatment focusing on individuals (Proenca, Rosko, & Zinn, 2000). However, when local tax authorities and community groups in the United States argued that hospitals, which failed to play a significant role in improving community health, should not receive tax exemptions (Russell, 1995), the impetus towards innovation that emphasizes comprehensive healthcare services beyond treating sick patients was created, leading to the birth of acute hospitals fulfilling various health needs in communities. One such innovation came from Proenca (1998), who defined CO as an organization‐wide generation, dissemination and response to community intelligence to address present and future community health needs. Proenca stated that higher degrees of CO among health service organizations would yield greater cost‐effectiveness, market shares and stakeholder satisfaction. Application of CO also reinforces the social justice and social accountability dimensions of public health (Gofin & Gofin, 2011), allowing public health practitioners to recognize and address social and environmental determinants of health as they obtain knowledge of the community and partnerships at community level (Muldoon et al., 2010). Thus far, several studies have examined the concept of CO in primary health care (PHC) institutions and among health professionals in countries other than the United States (Haggerty et al., 2007; Rodrigues & Witt, 2013; Tanabe & Yanagisawa, 2015).

## 2 BACKGROUND

Fiji currently faces a triple burden of health issues: communicable diseases, non‐communicable diseases (NCD) and injuries; this is a common trend observed in a growing number of low‐ and middle‐income countries (Wiseman et al., 2017). To provide a wide range of services to communities according to different health needs, the Ministry of Health and Medical Services (MOHMS) in Fiji has initiated the Wellness Fiji approach that forms the basis for reorienting PHC delivery to people in communities through expanded partnerships between health professionals and local communities (MOHMS, 2015a). As frontline PHC providers for the MOHMS, community health nurses (CHNs) in Fiji are responsible for providing both preventative care and clinical services to communities.

Several factors affect CHN task performance. First, under the recent health policy reform, clinical services have been decentralized to health centres. This shift has required CHNs to spend more time providing outpatient care in their facilities. Second, the geographical characteristics of Fiji pose significant challenges for the delivery of health services, as the population is dispersed over a large maritime region (MOHMS, 2015b). Third, CHNs usually visit communities alone, except on special occasions such as outreach team visits. CHNs in rural areas and outer islands are solely assigned to nursing stations and thus have limited opportunities to mimic and receive timely advice from senior CHNs and their supervisors. Senior nurses have developed competency in their work as they have learned to manage community health activities effectively through meaningful yet challenging experiences. These intangible experiences are difficult to convey verbally to their juniors, especially given the working situations of CHNs in Fiji, as described above. Identification of detailed characteristics and development of a CO framework among CHNs in Fiji will enable them to comprehend what sort of attention must be paid to meet community needs and manage community activities. Use of the CO framework as an education tool for supervisors will also improve communication between them and their CHNs, enabling CHNs to then implement CO‐based community activities that lead to superior understanding of community health needs, effective use of resources and increased community participation in activities. Such CO‐based activity management will improve community satisfaction and contribute to promote health, not just for the individual, but for the entire community.

### 2.1 Aims of the Study

The present study aimed to identify detailed characteristics of CO among CHNs in Fiji. In this study, we focused on individual CHNs. Therefore, characteristics of CO in this study included practices, knowledge and skills, values, attitudes and perceptions towards the generation, dissemination and response to community intelligence to address present and future community health needs. CHNs in Fiji were defined as those who worked in health centres and nursing stations and who were responsible for community health services in assigned zones and districts. The research question was “what characteristics of CHNs are related to the generation, dissemination and response to community intelligence to address present and future community health needs?”

### 2.2 Setting

Fiji has an area of more than 18,000 km^2^ and comprises 110 inhabited islands. As of 2015, it had the largest population of all Pacific island countries, at the population of 869,458 (Fiji Bureau of Statistics, 2016). Registered Nurses in Fiji are graduates of 3‐year pre‐service diploma of nursing programmes at the university or the nursing academy. They have also passed an examination by the Nursing Council of Fiji (MOHMS, 2014). Licences are renewed yearly by completing training (JICA, 2014).

There were 22.42 nurses per 10,000 population in 2015 (WHO, 2019), exceeding the World Bank minimum recommended rate (Bigbee, 2007). The nursing workforce is comprised of 76% staff nurses and 21% senior nurses with postbasic specialist skills, including managers, nurse practitioners and midwives. Registered Nurses provide a full range of services from PHC in health centres and nursing stations to acute care in hospitals. All health centres are staffed by either a doctor or a nurse practitioner with 1–20 nurses depending on the location and population. In addition, some large health centres have a pharmacy, laboratory, X‐ray set‐up, dental unit, health inspector, dietician and clinic nurses. Nursing stations located in the more isolated areas are staffed only by one CHN (Roberts et al., 2011).

Staff turnover is a major concern for nursing in Fiji (Fiji Times, 2009, 2011, 2015). Turnover is especially high among nurses working in remote areas, for various reasons that may include hard living environments, interpersonal relationship issues with people in the communities or supervisors and the desire for better quality education for their children (JICA, 2014). Internal migration from rural to urban areas is also problematic, as vacancies remain unfilled due to insufficient numbers of those with appropriate training available to serve; collectively, this results in uneven provision of healthcare services within country (Doyle & Roberts, 2013; JICA, 2014). In response to this situation, the Fiji government increased the number of new recruits beginning in 2014 (JICA, 2014). Given the current situation, in‐service training for newly recruited nurses, as well as those who wish to continue their career in MOHMS, is one of prioritized strategies for MOHMS to improve retention and motivation of CHNs in Fiji.

## 3 METHODS

### 3.1 Design

The present study was part of the chief investigator's doctoral research project, entitled “Community Orientation among Community Health Nurses in Fiji: Scale Development and Influencing Factors.” The present study employed a qualitative descriptive design. Semi‐structured individual interviews were conducted by the chief investigator between July–August 2015.

### 3.2 Participants

Individual interviews were conducted with participants of the following occupations: expert CHN (*N* = 9), novice CHN (*N* = 5), policymaker (*N* = 3) and community representative (*N* = 3). Inclusion criteria for these four occupations were as follows:

Expert CHN: a CHN supervisor or manager with more than 5 years of experience as a CHN or a community nursing lecturer at a school of nursing;
Novice CHN: a CHN with fewer than 5 years of experience who had submitted annual reports and competency assessment;
Policymaker: a national programme advisor, head of divisional health service office, or head of subdivisional health service office who had participated in national health policy planning and community representative: a community health worker, member of a health committee in a community, or peer counsellor.

All participants understood the purpose of the study and consented to participate. Participants were recruited by one of the investigators who was a director of nursing in the MOHMS at the time the interviews were conducted.

### 3.3 Data collection

Semi‐structured interviews were conducted by the chief investigator, who used an interview guide. Semi‐structured interviews were chosen because they can take advantage of the knowledge‐producing potential of interactions by allowing follow‐up on angles considered important by interviewees and also allow interviewers to become visible as a participant in the process (Brinkmann, 2018). General socio‐demographic data (age, education) and work‐related information (current and past positions) were collected prior to the interviews. The main theme of the interviews centred around parameters or characteristics of CO among CHNs, that is important practices, knowledge and skills, values, attitudes and perceptions of generation, dissemination and response to community intelligence to address present and future community health needs. In addition, expert CHNs were asked also to compare the more competent CHNs with less competent ones and identify underlying reasons for these differences. Novice CHNs were asked to describe any difficulties and obstacles pertaining to community activities. Policymakers were asked to describe the policy direction of community health services and the role of CHNs in community health activities. Community representatives were asked to describe the current health situation of their communities, any impressionable episodes that they experienced in relation to CHNs and community health activities and any requests pertaining to CHNs. All interviews were digitally recorded with permission from the participants. Notes were taken during the interviews and used for data analysis.

### 3.4 Analysis

Data were analysed using descriptive qualitative methods (Gregg, Asahara, & Yokoyama, 2007). All interview recordings and field notes were transcribed verbatim. Transcript accuracy was confirmed by the chief investigator. Phrases and sentences corresponding to and exemplifying the theme were extracted as initial codes. These words and sentences were merged into one sentence as final codes, which were then consolidated to form subcategories; these in turn were combined and contextualized into categories. Similarity and disparity were considered during the extraction process.

Each occupation comprised a unit for analysis. Categorizations were redefined through comparison by checking feasibilities, confirming policy directions and community expectations and analysing missing codes.

The chief investigator extracted codes and performed the categorization. Categorization drafts were created and revised multiple times. Analyses were repeatedly discussed and modified by all investigators, such that the finalized version reflected comments from the participants as well as public health and nursing researchers with experience working in Fiji.

The rigour of this study was ensured by the use of rich information obtained from participants of four different occupations. In addition, criteria developed by Guba and Lincoln (1985) were used to guide the analysis. To verify credibility, two experts in qualitative research were consulted about the accuracy, relevance and meaning of data. They also confirmed that the results reflected the voices of participants to ensure dependability. The results were further confirmed by two public health researchers who had experience working and researching in Fiji. Three participants, who were included in the interviews and shared their abundant experience, were asked to check and confirm the research results. To ensure transferability and confirmability, interviews were recorded and transcribed verbatim and multiple memos that were written to reflect the interview data were kept. The authors began the analysis immediately after each interview was completed and transcribed. Since no further codes were extracted after analysing the 20th interview, saturation was considered to have occurred.

### 3.5 Ethical considerations

All participants were informed of the purpose, procedure, potential contributions of the study, confidentiality and their right to refuse to participate. To ensure anonymity of study data, participants were assigned ID numbers and all potentially identifying information was coded. Written informed consent was obtained from all participants and their department heads prior to the interviews. This study was approved by the Fiji National Health Research Ethics Review Committee (2014.122.MP) and the Institutional Review Board for Research Ethics of Aichi Prefectural University (26APU‐UGA2‐15).

## 4 RESULTS

### 4.1 Participant characteristics

Table 1 summarizes the characteristics of participants. Expert CHNs (*N* = 9) ranged in age from the 30s–60s and each had 5–20 years of experience as a CHN and a supervisor. Six had obtained both a bachelor's degree in nursing science and an associate's degree in nursing. Novice CHNs (*N* = 5) were in their 20s and 30s and had 1–3 years of experience as a CHN. All had an associate's degree in nursing. Policymakers (*N* = 3) were all in their 50s, all had obtained a bachelor's degree in medicine, and two had obtained a master's degree in public health. One of the community representatives was in her 20s, and two were in their 60s. Two of the community representatives were college graduates and had associate's degrees. One community representative was a university graduate and had a bachelor's degree. All 20 participants were natives of Fiji.

**Table 1:** Participant characteristics

Group	Group			
	Expert CHN (9)	Novice CHN (5)	Policymaker (3)	Community representative (3)
Age (years)				
Average ± *SD*	49.33 ± 6.38	28 ± 2.53	51.33 ± 1.25	53 ± 16.99
20–29		3		1
30–39	1	2		
40–49	2			
50–59	5		3	
60 and above	1			2
CHN/superior experience (years)	5–20+	1–3	‐	‐
Education				
Associate's degree in Nursing/others	8	5		2
Bachelor of Nursing Science/others	7			1
Diploma in Public Health	5			
Bachelor of Medicine			3	
Master of Public Health	1		2	

### 4.2 Community orientation of community health nurses in Fiji

A wide range of information concerning CO among CHNs in Fiji was extracted from the interviews. Most codes and subcategories were extracted from expert CHNs and so this group was set as the main group for analysis. None of the final codes from expert CHNs contradicted those of other groups. The codes extracted from the groups other than expert CHNs were added to the appropriate categories of expert CHNs if they were not included in those for expert CHNs. Extracted codes of the four groups were consolidated into 57 final codes (Table 2). The analysis ultimately revealed three main categories: Trusting Relationships, Commitment and Activity Management.

**Table 2:** Community orientation among community health nurses in Fiji

Subcategories	Final codes
Category: Trusting relationships	
Pay careful attention to community member acceptance	Show respect and reverence to community members
	Communicate in an approachable and friendly manner
	Visit communities frequently even in addition to professional work purposes
	Participate in as many social activities as possible
	Review conduct and attitude towards community members on a daily basis
Be reliable and trustworthy towards community members	Provide services in a responsible and sincere manner
	Confer with colleagues and supervisors before returning to the individuals if faced with any uncertainty about how to answer questions
	Fulfil any promises once made
	Demonstrate willingness to contribute and be useful to community members
	Contribute to social activities
	Maintain confidentiality with community members’ information
	Skilful clinical care earns the trust of community members
Be expressive and sensitive to the culture and lives of community members	Empathize with the feelings of community members
	Show respect for the cultures and traditions of communities and try to live in a manner similar to others in the community
	Think in a way that reflects community perceptions and understand their situations
Let community members become familiar with CHNs	Explain roles and tasks of CHNs to community members
	Be aware of the community's curiosity towards CHNs
	Show personalities and share private lives
	Express own thoughts and opinions to community members
Strengthen relationships with stakeholders and resource people to collaborate on activities	Approach stakeholders and resource people to create and maintain relationships
	Actively inform resource people about issues of concern
Category: Commitment	
Recognize responsibilities of CHNs to community members	Perceive a sense of mission when an issue arises
	Make continued efforts towards resolution, even if services are unavailable through any governmental and non‐governmental organizations
	Try best to meet client needs with limited resources
	Serve as role models for community members and junior CHNs
	Avoid bringing private issues to the workplace
	Maintain honesty in reporting and record‐keeping
Professional development	Learn from supervisors and colleagues
	Apply the concepts, models and methods learned at school and in workshops to their activities
	Assess own weaknesses and limitations
Promote teamwork	Support supervisors and colleagues
	Observe supervisors and colleagues to determine whether assistance can be provided
Category: Activity management	
Learn about the community from and with the community	Take interest in promoting and maintaining health of communities
	Demonstrate curiosity about the communities
	Seek support from community members and stakeholders to collect information
	Observe districts by setting
	Collect information about daily living conditions, norms that exist in communities, community members and relationships among them and groups and organizations with whom CHNs might collaborate
	Try to find out strengths as well as issues underlying health problems
	Pay careful attention to issues that might affect minorities and vulnerable groups
Build consensus with community members and stakeholders	Apply medical and nursing knowledge to examine the collected information
	Determine health problems, target groups and backgrounds affecting health problems
	Present analysis to community members, especially to health committees in the villages and workplaces
	Discuss solutions until a mutual consensus is achieved when community member priorities differ from those of CHNs
	Effectively and persuasively use presentation and negotiation skills
Lay groundwork for community activities	Review available assets
	Communicate with resource people to collaboratively set targets and objectives and to plan activities
	Consider whether to target small populations or a whole community depending on health problems and assets
	Consult with and submit proposals to supervisors
	Spend time preparing details and materials for community activities
	Designate roles, as well as motivate and train resource people to implement activities rather than assuming leadership over activities
	Prepare for activities together with heads of the villages and community health workers
Actively partner with community members in the activities	Experience community activities as a participant in order to share experiences of other participants from communities
	Ask participants for their feedback after the activities
	Take on the responsibility of monitoring and follow‐up after the activities, while delegating various responsibilities to community members
	Identify imperceptible changes in behaviour and in perceptions of participants and other community members after the activities
	Record and report on the activities
	Identify lessons learned and appreciate the sense of achievement and self‐esteem that come from accomplishing the activities

### 4.3 Trusting relationships

It is essential for CHNs to establish close relationships with community members so that the latter would voice their concerns voluntarily to CHNs. Establishing and maintaining mutually Trusting Relationships that promote empowerment of communities require careful attention by CHNs, as demonstrated by five subcategories: pay careful attention to community member acceptance, be reliable and trustworthy towards community members, be expressive and sensitive to the culture and lives of community members, let community members become familiar with CHNs, and strengthen relationships with stakeholders and resource people to collaborate on activities.

#### 4.3.1 Pay careful attention to community member acceptance

Community health nurses show respect and reverence to community members and communicate in an approachable and friendly manner so that community members are willing to talk to the CHNs. They take active and intentional steps to visit communities frequently in addition to their professional work purposes so as to maintain these relationships. They also try to participate in as many social activities as possible to make their presence known in the community and to communicate with various community members. They also review their conduct and attitude towards community members daily.

#### 4.3.2 Be reliable and trustworthy towards community members

Community health nurses work to provide services in a responsible and sincere manner, such as by responding to inquiries and requests in an accurate and timely manner. They work to confer with colleagues and supervisors before returning to the individuals if they face any uncertainty about how to answer questions. They take steps to fulfil any promises once made. However, situations sometimes create difficulties for these endeavours due to poor weather or unexpected orders from their affiliated department. They strive to demonstrate their willingness to contribute and be useful to community members, such as by providing valuable information, particularly at the first meeting, because they believe that their first impression is long‐lasting. They also try to contribute to social activities such as fund‐raising events and funerals. They are careful to maintain confidentiality of community members’ information. They believe that skilful clinical care earns the trust of community members, which then paves the way for health promotion activities to flourish in communities.

#### 4.3.3 Be expressive and sensitive to the culture and lives of community members

Community health nurses try to empathize with the feelings of community members. They work to show respect for the cultures and traditions of communities and try to live in a manner similar to others in their community, such as by eating local food and speaking the local dialect if they were born and raised in a different area. They try to think in a way that reflects community perceptions and understand their situations rather than criticize community members who do not follow the CHN's advice.

#### 4.3.4 Let community members become familiar with CHNs

Community health nurses explain their roles and tasks to community members so that community members can understand what to expect from CHNs. They have an awareness of the community's curiosity towards CHNs. This is especially true for those who are newly assigned young nurses in rural areas, and as a result, they try to reply to any personal questions and comments. They work also to show their personalities and share their private lives such as families and interests, knowing that finding common ground creates a closeness and sense of connection with each other. They try to express their own thoughts and opinions to community members.

#### 4.3.5 Strengthen relationships with stakeholders and resource people to collaborate on activities

Community health nurses intentionally approach stakeholders and resource people to create and maintain relationships. They contact these individuals by phone and visit them when they are nearby. In addition, they make sure to actively inform the resource people about issues of concern.

### 4.4 Commitment

Community health nurses strongly perceive Commitment to be a key organizational principle as well as a critical component of their professionalism. Recognize responsibilities of CHNs to community members, professional development and promote teamwork were subcategories of this category.

#### 4.4.1 Recognize responsibilities of CHNs to community members

Community health nurses perceive a sense of mission when an issue arises and respond to it immediately. They also make continued efforts towards resolution, even if services are unavailable through any governmental and non‐governmental organizations. One novice CHN expressed her unsolved case as follows:

I contacted other government organizations but have not found any solutions yet. I realized that there is no available support system for him. However, I cannot leave him. Nobody will help him if I don’t.

Community health nurses in Fiji face limited means of transportation, communication and material resources, but try their best to meet client needs with the limited resources. They try to serve as role models for community members and junior CHNs and avoid bringing private issues to the workplace. They strive to maintain honesty in their reporting and record‐keeping. For example, they do not hide information, but rather report everything, including issues such as client complaints or increased numbers of defaulters that represent an inconvenience to them.

#### 4.4.2 Professional development

Community health nurses are eager to learn from supervisors and colleagues, as exemplified by the fact that they frequently seek advice and try to learn about community activity methods from colleagues and other sources such as workshops and media. They apply the concepts, models and methods to their activities. One novice CHN described her efforts as follows:

I like to apply concepts and models such as the Gibbs Trust Model, motivational interviews and Plan‐Do‐See that I learned at school and in workshops. When I talk with community members, I analyze them using these concepts so that I can understand their stages and what I should and should not do. Also, when getting ideas such as zumba (exercise program) and healthy baby campaigns from media and colleagues, I am enthusiastic and keen to try those in my community.

They regularly assess themselves for weaknesses and limitations, to strengthen and empower themselves and plan their next steps accordingly.

#### 4.4.3 Promote teamwork

Community health nurses work to support their supervisors and colleagues. They actively observe their colleagues and supervisors to determine whether they could provide any assistance.

### 4.5 Activity management

Community health nurses follow every process of a community activity together with community members and stakeholders. CHNs generate, disseminate and respond to community health needs by learning about communities from and with communities, build a consensus with community members and stakeholders by sharing collected information, lay groundwork for the community activities and are active partners with community members in the activities.

#### 4.5.1 Learn about the community from and with the community

Community health nurses take interest in promoting and maintaining the health of communities in addition to providing clinical services to individual clients. They demonstrate curiosity about the communities and do not collect information independently but instead would seek support from community members and stakeholders with whom they might collaborate in community activities. They observe their districts by setting such as mother groups, youth groups and schools formed as a unit. During daily routine services, they aim to collect information about daily living conditions, norms that exist in communities, community members and relationships among them and groups and organizations with whom CHNs might collaborate. They try to find strengths as well as issues underlying health problems. CHNs pay careful attention to issues that might affect minorities and vulnerable groups.

#### 4.5.2 Build a consensus with community members and stakeholders

Community health nurses work to apply medical and nursing knowledge to examine the collected information. For example, they use comparisons to explain situations both effectively and persuasively. They determine health problems, target groups and backgrounds affecting health problems. They then present their analysis to community members, especially to health committees in the villages and workplaces. They discuss solutions until a mutual consensus is achieved when community member priorities differ from those of CHNs. To achieve this, they effectively and persuasively use presentation and negotiation skills.

#### 4.5.3 Lay the groundwork for community activities

Community health nurses review available assets: facilities, human resources, organizations, groups and funds. They actively work to communicate with resource people collaboratively to set targets and objectives and to plan activities. They consider whether to target either small populations or a whole community depending on the health problems and assets. They voluntarily consult with and submit proposals to their supervisors. They spend time preparing details and materials for community activities. They work to designate roles, as well as motivate and train resource people to implement activities rather than assume leadership over activities. They prepare for activities together with heads of the villages and community health workers, such as by coordinating times so that schedule conflicts are avoided.

#### 4.5.4 Actively partner with community members in the activities

Community health nurses experience community activities together with community members to share experiences of other participants from communities. They ask participants for their feedback after the activities. Although CHNs delegate various responsibilities to community members, they take on the responsibility of monitoring and follow‐up after the activities. They try to identify imperceptible changes in behaviour and in perceptions of participants and other community members after the activities. They record and report the activities, identify lessons learned and appreciate the sense of achievement and self‐esteem that come from accomplishing these activities.

## 5 DISCUSSION

The central aim of the present study was to identify detailed characteristics of CO among CHNs in Fiji; these were defined as important practices, knowledge and skills, values, attitudes and perceptions towards generation, dissemination and response to community intelligence to promote community health. We identified three main categories that describe this particular theme: Trusting Relationships, Commitment and Activity Management.

In terms of Trusting Relationships, CHNs made continuous efforts and paid careful attention to individuals in the communities. A community comprises multiple persons as an interactional unit (Schultz, 1987). Gottlieb, Feeley, and Dalton (2006) noted that collaboration in community health activities requires nurses and individuals in the community to be mutually open and respectful, to show tolerance for another person's beliefs and to understand oneself, others and situations from another person's perspective. These perceptions and attitudes were observed in the final codes for Trusting Relationships. Zeidler (2011) noted that established relationships with individuals and nurses pave the way for a mutual willingness to ask and respond; consistent with this, Trusting Relationships is a prerequisite for information collection and responding to health needs. CHNs also intentionally strengthened relationships with stakeholders and resource people to collaborate on activities because the CHNs aimed to expand their relationships beyond just the individual in the community, to the community as a whole, through stakeholders and resource people who could influence other community members.

During the interviews, many participants (not only CHN supervisors and policymakers but also novice nurses) described characteristics of CO using the term “commitment.” The interviewer asked participants to elaborate on this point, and the authors carefully analysed their descriptions, which were sorted into three subcategories: recognition of CHNs’ responsibilities to community members, professional development and promoting teamwork. Organizational commitment is defined as a psychological link between an employee and his or her organization that makes it less likely that the employee will voluntarily leave the organization (Allen & Meyer, 1996). Individuals who find greater commonality between their personal beliefs and their profession are more likely to care about the future of their profession and remain in it (Lachman & Aranya, 1986). Such individuals commit to the organizational goals and values more and increase their job involvement to improve job effectiveness and productivity (Meyer & Herscovitch, 2001; Mowday, Steers, & Porter, 1979). CHNs found personal identification as CHNs and morally recognize their roles as CHNs. Hence, they exert their utmost effort to provide effective care for community members. Such CHNs continue to develop their professional competencies as CHNs. Promoting teamwork is a normative commitment that reflects an obligation to the organization and efforts to maintain good relationships with colleagues and supervisors. Commitment of CHNs is an antecedent to the empowerment process for community health activities (Rodwell, 1996), thus a crucial domain for CO as its goal is enhancing service quality and improving access to care.

In terms of Activity Management, CHNs involved community members in each step of the community activities. Management of community activity is a process requiring nurses to assess, plan, implement and evaluate as part of a systematic decision‐making process characterized by cognition, client‐centredness and directional goals (Clark, 1996). Community members are neither mere data sources nor targets for intervention. Community members determine what healthcare services should be provided, consistent with PHC principles (Rifkin, 1986). True empowerment in a community can occur only when its members have the knowledge required to assess their situation and take action to affect change (Hancock & Minkler, 2012). Therefore, community participation to generate community intelligence represents the first part of Activity Management, both for empowerment of community members and for assessing community health needs. CHNs involved influential community members in the planning and implementation of community activities. While non‐official community leadership is less obvious and may be more difficult to detect, this leadership often exerts more influence, power and control over community action and decision‐making than that of official leaders (Clemen‐Stone, Eigsti, & McGuire, 1995). Based on information about community resources and their relationships gleaned by the CHNs from community members, CHNs both motivated and trained such non‐official leaders according to their abilities and interest during the planning process. The present study confirmed the importance of conducting this process in the context of CO.

**Figure 1:**
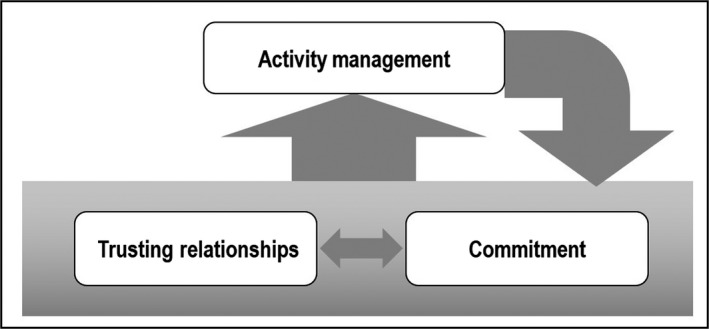
Conceptual framework of community orientation among community health nurses in Fiji

In terms of the sequence and relationships among the three categories, Trusting Relationships and Commitment are antecedents for Activity Management, as discussed earlier. Identifying needs and responding to people in a committed way creates a trusting relationship (Yamashita, Miyaji, & Akimoto, 2005). By gaining a more comprehensive understanding of community member lifestyles based on Trusting Relationships, the CHNs increased their Commitment towards their tasks, which further motivated them to respond sincerely to community health needs. In this manner, Commitment and Trusting Relationships are interrelated, serve as foundations for CO and promote and facilitate Activity Management. Reflection and self‐accomplishment of experiences during Activity Management further strengthen Commitment and Trusting Relationships. The conceptual framework of CO among CHNs in Fiji is proposed accordingly (Figure 1).

During interviews, policymakers emphasized the importance of targeting specific populations, while novice CHNs opined that access to community health activities should not be limited to certain individuals, but rather be open to any community members. Rose (2001) noted two approaches: a high‐risk strategy seeking to protect susceptible individuals identified as exhibiting an elevated risk for adverse health outcomes, and a population strategy that targets the entire population and seeks to control the causes of incidences. CHNs could benefit from combining or selecting one of the two approaches, depending on community health needs. Generation of community intelligence is important to determine a strategy for executing community activities and their superior understanding of community health needs results in selecting an appropriate strategy, the latter of which would increase the effectiveness of activity management.

The study also confirmed the importance of equity in Fiji by extracting the final code: paying careful attention to issues that might affect minorities and vulnerable groups. Gender inequality, poverty and ethnicity issues are persistent challenges in Fiji that limit equity in healthcare access (Chattier, 2005; Tulloch, 2011). Reduction of exclusion and social disparities in health is a central theme for PHC and Universal Health Coverage, which MOHMS has adopted and included as one of its guiding principles (MOHMS, 2015a; WHO, 2015). The present findings suggest that CHNs should continue to generate a lively discussion surrounding this issue and incorporate focus on these issues into their main concerns to promote health for the whole community regardless of gender, economic status or ethnicity. Careful attention to these matters will expand access to community intelligence that was previously lacking. A strong understanding of the diversity among community members will also improve their response to community health needs. Consequently, coverage of health services will be expanded, with greater satisfaction.

To our knowledge, the present study is the first to explore detailed characteristics of CO among PHC professionals. The study examined perspectives not only of CHNs, their superiors and faculty members, but also of policymakers and community members that ensured enough variation for extracting the topics. There were several notable limitations in this study. First, environmental and ethnic contexts can vary considerably across communities in Fiji. There are discrepancies between health situations and cultures in densely populated cities and those in sparsely populated rural villages. The actual community activity management differs by community. Therefore, some of the present study findings may not be applicable to all areas. Second, this study did not target any specific illness or health needs such as NCD, adolescent health and mental health but covered community health needs in general. The conceptual framework and subcategories are applicable in all areas and for all health programmes that are implemented in communities by CHN; however, the degree of importance and relevance of detailed characteristics that are the final codes may differ among areas and the health programmes. Further investigation is needed to identify detailed characteristics of CO in particular settings (e.g. rural and urban areas) and particular community health needs. Third, this study is based on individual interviews with CHNs, health professionals and volunteer community health workers, and thus, the data may be fairly subjective. Other participants may have different views, and other modes of data collection such as participant observations and report analyses would likely yield different findings pertaining to CO among CHNs in Fiji. Future studies should employ other forms of data collection and should carefully examine the findings from multiple perspectives, especially those of community members who require CHN support and of health professionals other than physicians.

Further research is required to develop a measurement tool and explore factors that influence CO. The measurement tool will enable CHNs to assess the degree of CO implemented in their work and enable policymakers in MOHMS to identify influencing factors and develop a supportive environment for CHNs.

## 6 CONCLUSIONS AND PRACTICAL IMPLICATIONS

The present study extracted three main categories, 12 subcategories and 57 final codes with regard to the concept of CO among CHNs in Fiji. CHNs work continuously to create Trusting Relationships and increase their Commitment towards community members and organizations. Trusting Relationships and Commitment are interrelated and serve as foundations for CO, but also promote and facilitate Activity Management. Reflection and self‐accomplishment of CHN experiences during Activity Management further strengthen Commitment and Trusting Relationships.

By referring to this framework, CHNs will be able to understand important practices, values and perceptions of CO, which can be used as reflection materials for CHNs, coaching materials for supervisors and educational materials for students in community nursing programmes.

## CONFLICT OF INTEREST

The authors declare no financial or other conflicts of interest.

## AUTHOR CONTRIBUTIONS

ST and SY were responsible for the study conception, design, and drafting of the manuscript. ST performed the data collection. All authors performed the data analysis. SY made critical revisions to the manuscript and supervised the study. All authors read and approved the final manuscript.
